# Bridging Generations: Redefining Age-Friendly Universities Through Innovation and Inclusion

**DOI:** 10.1093/geroni/igaf122.1004

**Published:** 2025-12-31

**Authors:** Zhuolin Chen, Long Ki Fung, Wai Tong Lee, Helene Fung, Jean Woo

**Affiliations:** The Chinese University of Hong Kong, Hong Kong, China (People’s Republic); The Chinese University of Hong Kong, Hong Kong, China (People’s Republic); The Chinese University of Hong Kong, Hong Kong, China (People’s Republic); The Chinese University of Hong Kong, Hong Kong, China (People’s Republic); The Chinese University of Hong Kong, Hong Kong, China (People’s Republic)

## Abstract

The Chinese University of Hong Kong (CUHK) has been recognized as an Age-Friendly University (AFU) for its comprehensive and innovative approach to integrating older adults into academic and campus life. In preparation for submitting our AFU application, CUHK conducted a thorough assessment of existing programs, policies, and practices to ensure alignment with the AFU principles. This included engaging stakeholders across the university and community to identify areas for enhancement. Guided by AFU principles, CUHK actively involves older adults in teaching, research, and campus activities, while promoting public discourse on aging-related issues. The university offers specialized programs in gerontology and its Jockey Club Institute of Ageing, leads research and community projects on critical areas such as developing tailor-made framework and implementing activities under the principles of integrated care of older people, tackling the impact of climate change on older people, and using human-centric AI and robotics technology for geriatric care. Initiatives like the Actionable Social Knowledge (CU-ASK) program in the Motivation and Emotion Laboratory exemplify CUHK’s commitment to bridging generational gaps and developing evidence-based solutions. Additionally, intergenerational projects such as the Age-Friendly City program and Elder Academy foster mutual understanding and collaboration between generations. Our survey findings on the AFU underscore the critical importance of recognizing the range of education needs of older adults. Moving forward, CUHK will prioritize the development and integration of health and wellness programs specifically designed for older adults and foster intergenerational learning opportunities, thereby enhancing the university’s commitment to inclusivity and lifelong learning.

